# Halophytic *Hordeum brevisubulatum* HbHAK1 Facilitates Potassium Retention and Contributes to Salt Tolerance

**DOI:** 10.3390/ijms21155292

**Published:** 2020-07-25

**Authors:** Haiwen Zhang, Wen Xiao, Wenwen Yu, Ying Jiang, Ruifen Li

**Affiliations:** 1 Beijing Key Laboratory of Agricultural Genetic Resources and Biotechnology, Beijing Agro-Biotechnology Research Center, Beijing Academy of Agriculture and Forestry Sciences, Beijing 100097, China; hwzhang0422@hotmail.com (H.Z.); xiaowen7821@163.com (W.X.); 18800109890@163.com (W.Y.); jiangying198212@126.com (Y.J.); 2 Beijing Key Laboratory of Plant Gene Resources and Biotechnology for Carbon Reduction and Environmental Improvement, College of Life Sciences, Capital Normal University, Beijing 100048, China

**Keywords:** HAK/KUP/KT transporter, K^+^-uptake, salt-tolerance, K^+^/Na^+^ homeostasis, site mutation

## Abstract

Potassium retention under saline conditions has emerged as an important determinant for salt tolerance in plants. Halophytic *Hordeum brevisubulatum* evolves better strategies to retain K^+^ to improve high-salt tolerance. Hence, uncovering K^+^-efficient uptake under salt stress is vital for understanding K^+^ homeostasis. HAK/KUP/KT transporters play important roles in promoting K^+^ uptake during multiple stresses. Here, we obtained nine salt-induced HAK/KUP/KT members in *H. brevisubulatum* with different expression patterns compared with *H. vulgare* through transcriptomic analysis. One member HbHAK1 showed high-affinity K^+^ transporter activity in *athak5* to cope with low-K^+^ or salt stresses. The expression of HbHAK1 in yeast Cy162 strains exhibited strong activities in K^+^ uptake under extremely low external K^+^ conditions and reducing Na^+^ toxicity to maintain the survival of yeast cells under high-salt-stress. Comparing with the sequence of barley HvHAK1, we found that C170 and R342 in a conserved domain played pivotal roles in K^+^ selectivity under extremely low-K^+^ conditions (10 μM) and that A13 was responsible for the salt tolerance. Our findings revealed the mechanism of HbHAK1 for K^+^ accumulation and the significant natural adaptive sites for HAK1 activity, highlighting the potential value for crops to promote K^+^-uptake under stresses.

## 1. Introduction

Potassium represents 2.6% of the weight of the Earth’s crust. However, dissolved K in soil—as the only fraction directly available to plant—is deficient, especially in saline–alkali land and arid land [1]. Retaining potassium under stress conditions in plants has emerged as novel and essential mechanisms for plant survival [2,3]. Studying the mechanisms of highly efficient uptake of potassium from the soil in roots is critical for the growth and production of crops.

As ion fluxes control ion concentration, the HAK/KUP/KT (high affinity K^+^/K^+^ uptake proteins/K^+^ transporter) proteins function as K^+^-transporters and the major contributor for K^+^ nutrition in K^+^ depleted soil [4]. This family is widespread in all of the plant kingdom; 913 HAK/KUP/KT sequences have been identified in 46 plant genomes, with an uneven distribution between dicotyledonous and monocotyledonous species [5]. The information available on crop genomes shows a richness of HAK/KUP/KT transporters (about 27 members) compared with dicotyledonous plants (13 members in *Arabidopsis*), which indicates the important physiological roles of such transporters in crops. Their proteins possess 10–15 transmembrane (TM) domains and are divided into 5 clusters [5]. Genes in cluster I are the most functionally revealed, including classical *AtHAK5*, *HvHAK1*, *OsHAK1*, *OsHAK5*, *ThHAK1*, *SlHAK5*, *CcHAK1* and so on [6,7,8,9,10,11,12]. They all have a high-affinity K^+^ transport feature that allows plant to thrive under low-K^+^ (<1 mM) conditions. In fact, K^+^ uptake in plants also demands these HAK-type transporters under multiple stresses such as salinity, Cs^+^ -polluted or drought soils [4]. One reason is that higher external Na^+^, Cs^+^ or drought indirectly reduces soluble K^+^ concentration in soil that leads to K^+^ deficiency [13,14,15]. On the other hand, accumulations of Na^+^ or Cs^+^ in cells causes ion imbalances with a reduced K^+^/Na^+^ or K^+^/Cs^+^ ratio that inhibits plant growth [16,17]. In crops, the loss of *OsHAK1* in rice leads to about 50–55% lower K^+^ uptake than wild types in K-starved environments, and causes sensitivity to drought and salt [8,18]. OsHAK1, OsHAK5, OsHAK16 and OsHAK21 can coordinately regulate Na^+^/K^+^ homeostasis and the membrane potential of root cells under salt conditions [4,8,9,18,19]. A genome-wide association (GWAS) study in *Zea mays* L. showed ZmHAK4-mediated shoot K^+^/Na^+^ homeostasis for improving salt tolerance, conferring the natural variation of this gene [20]. Therefore, under saline conditions, K^+^ nutrition largely depends on HAK/KUP/KT system, affecting agriculture worldwide.

Saline land occupies more than 20% of irrigated soil. It reduces crop productivity because most crops are glycophytes [21]. As barley is the most salt-tolerant species among crops, several wild *Hordeum* species are halophytes [22,23]. *Hordeum brevisubulatum* is perennial among these halophytic species and could be used as a saline-tolerant grass for soil improvement in North China [24]. Our previous reports have shown its remarkable salt tolerance due to low Na^+^ accumulation and stable K^+^ content compared with barley [25,26]. Although the genome of *H. brevisubulatum* is unpublished, a transcriptomic analysis could provide clues for gene identification and understanding of salt-tolerance molecular mechanism. We extracted and identified 13 HAK/KUP/KT members from salt-treated transcriptomic data. Because of the different expression patterns between these genes in *H. vulgare* and *H. brevisubulatum*, we focused on functional study of HbHAK1. As the genus *Hordeum* belongs to *Triticeae*, the sequences of its functional genes share many similar sequences with barley and wheat [26]. HbHAK1 showed better transport activity than barley HvHAK1, though only 15 amino acids residues were different between them. Based on the 15 amino acids, further studies revealed C170 and R342 conserved amino acids significantly involved in the K^+^ selectivity and transport activity under extremely low-K^+^ conditions (10 μM), and A13 directly affect the K^+^ uptake under high external Na^+^ condition. Our study significantly revealed the natural adaptive sites of HbHAK1 were the key residues for improving HAK1-type activity, highlighting the potential value for crops to promote K^+^ uptake under stresses.

## 2. Results

### 2.1. HAK/KUP/KT Proteins Were Identified According to Transcriptomic Analysis

**Figure 1:**
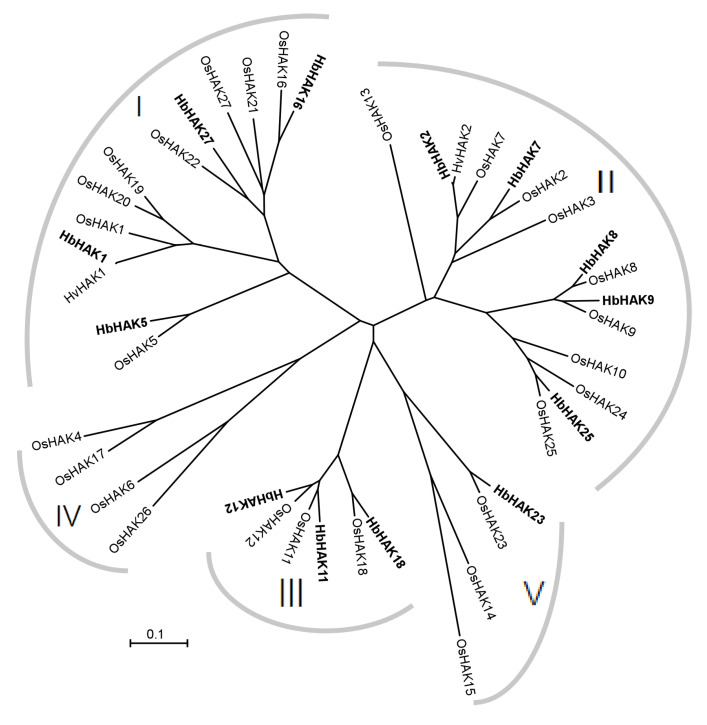
Phylogenetic relationships between identified HAK/KUP/KT proteins in *H. brevisubulatum* and all the HAK/KUP/KT proteins in rice. Phylogenetic tree was generated with MEG5.0 software (http://www.megasoftware.net/) using the neighbor-joining method. The scale indicates the genetic distance.

Our previous reports revealed that *H. brevisubulatum* plants could maintain a relatively stable level of K^+^ content under saline conditions and the numbers of upregulated genes of potassium transporters greatly exceeded that in *H. vulgare*, which showed obvious K^+^ loss under saline conditions [26]. As these large amounts of K are taken up from soil and then are re-translocated and cycled, we performed Illumina RNA sequencing (RNA-seq) on a time series (0, 1, 6 and 24 h) of the roots of 21-day-old *H. brevisubulatum* and *H. vulgare* plants under 350 mM NaCl treatment. We searched the potential HAK/KUP/KT transporters in PacBio isoform sequencing (Iso-seq) data. Finally, 13 genes with intact coding DNA sequences were obtained and named following the homologous genes in rice and barley. These proteins contained about 708–945 amino acids and were predicted to contain 11–14 trans-membrane regions with a long tail in the C-terminal. All these proteins were localized in the plasma membrane, which were analyzed using TargetP tool (http://www.cbs.dtu.dk/services/TargetP/) (Table 1). Based on the alignment of these 13 proteins in *H. brevisubulatum* and the HAK/KUP/KT proteins in rice, a phylogenetic tree was constructed (Figure 1). HAK/KUP/KT members can be divided into 5 clusters, as crop genomes contains all the 5 clusters while the *Arabidopsis* has only 4 clusters [5]. The 13 proteins were localized in 4 clusters. No genes belonged to Cluster IV, in detail, as 4 in cluster I (8 in rice), 5 in cluster II (9 in rice), 3 in cluster III (3 in rice) and 1 in cluster V (3 in rice) (Figure 1).

### 2.2. HbHAK1 Was Upregulated by Salt Stress in Roots with the Differential Expression Patterns

**Figure 2:**
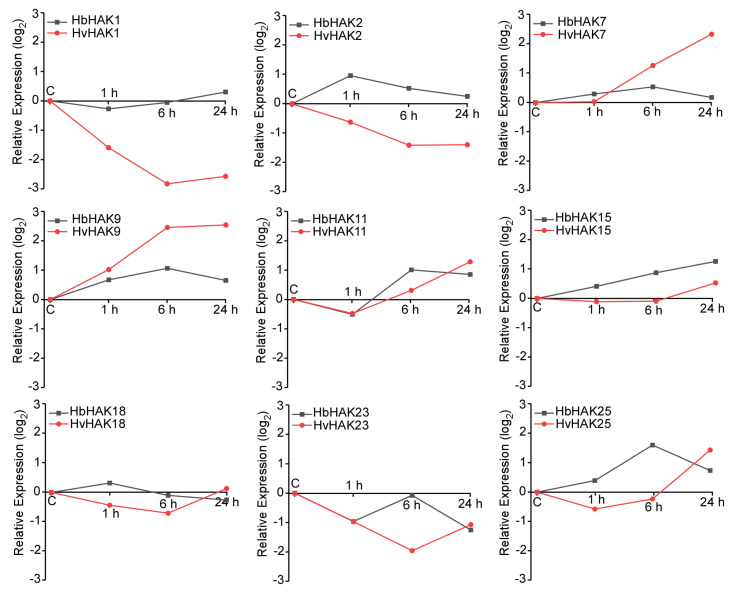
Selected HAK/KUP/KT genes with different expression pattern from barley. The expression level in different time course was compared with no-NaCl treatment control. The data were using a criterion of absolute log2(fold-change) of the relative expression levels.

In order to explore the mechanism of efficient K^+^ uptake in *H. brevisubulatum*, we extracted the expression information of 13 identified HAK/KUP/KT proteins from transcriptomic data of root tissues. The homologous genes coding these 13 HAK/KUP/KT proteins in *H. vulgare* from transcriptomic data were also extracted. Comparing these genes in *H. brevisubulatum* and *H. vulgare*, 9 members with different expression patterns were identified (Figure 2). Interestingly, *H. brevisubulatum* and *H. vulgare* showed the definitely opposite expression patterns in *HbHAK1* and *HbHAK2* under salt stress, as the *HbHAK1* and *HbHAK2* were upregulated while the *HvHAK1* and *HvHAK2* were obviously negative-induced under high-salt conditions (Figure 2). Notably, *HvHAK1* showed obvious downregulated expression pattern with about 85% lower than normal conditions after 6 h salt treatment, while the expression level of *HbHAK1* was stable—even 50% higher than normal conditions at the 24-h point. HbHAK1 and HvHAK1 all belonged to Cluster I subfamily. Many genes in this cluster are also involved in salt tolerance. Therefore, revealing HAK1-type K^+^ transporters in halophytic plants may improve our knowledge of understanding K nutrition from soil under saline conditions.

### 2.3. HbHAK1 Worked as a High-Affinity K^+^ Transporter on the Plasma Membrane and Involved in Salt Tolerance

**Figure 3:**
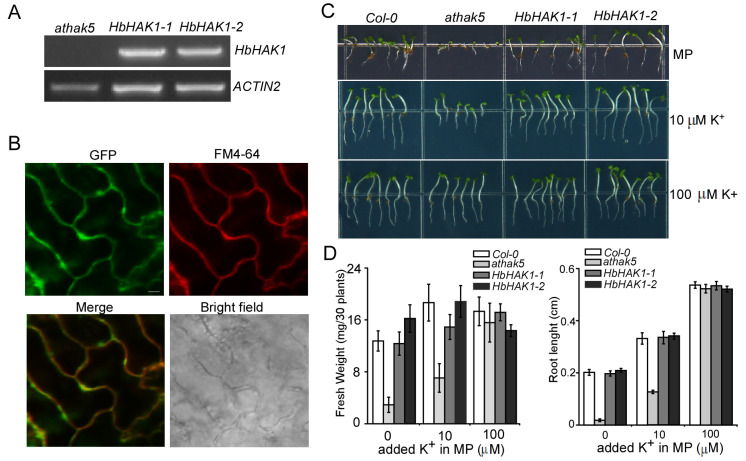
The expression of *HbHAK1* rescued the growth inhibition of *athak5* under low-K^+^ condition. (**A**) RT-PCR analysis of the HbHAK1 mRNA level in *athak5* and two *HbHAK1* transgenic lines (*HbHAK1*-*1*, *HbHAK1*-*2*); (**B**) Leaf epidermal cells in transgenic *athak5* plants expressed HbHAK1-GFP. The GFP signal (green) is on the top left; the plasma membrane stained with FM4-64 (red) is on the top right; a merge (green and red) is on the bottom left; the bright field microscope image is on the bottom right. Scale bar = 20 μm; (**C**) The wild type, *athak5*, and two *HbHAK1*-*GFP* transgenic lines in *athak5* (*HbHAK1*-*1*, *HbHAK1*-*2*) were grown for 7 days on MP medium with various K^+^ added; (**D**) Fresh weight and root length of plants in (**A**). Each bar represents the mean fresh weight (*n* = 3) of 30 seedlings, and the mean root length of 30 seedlings from three independent experiments. The data represent the mean ± SD.

The *Arabidopsis athak5* mutant showed growth defects in the low-K^+^ environment while the growth can be gradually recovered with the environmental K^+^ content increasing [27]. Therefore, we developed the fusion construct *HbHAK1*::GFP under the control of CaMV 35S promoter and transformed it into *athak5*. The *HbHAK1* transcription was detected in the transgenic lines of *athak5* (Figure 3A). As shown in Figure 3B, GFP fluorescence on the epidermal cells of leaves in transgenic lines was particularly observed on the plasma membrane, coinciding well with the fluorescence of FM4-64 stain (Figure 3B).

To examine the function of HbHAK1, we selected two transgenic lines in the *athak5* mutant background to access the growth of plants under low-K^+^ conditions or high-salt conditions. The *athak5* mutants showed severe growth defect on MP medium without K^+^ added, including root growth and cotyledon development, however, the expression of *HbHAK1* was obviously rescued this phenotype, performing the same growth as wild type (Figure 3C). With the external K^+^ content increasing, the growth of *athak5* was recovered, and this phenotype gradually became indistinguishable from transgenic lines and wild type. The root length and fresh weight of *athak5* were at the same level with transgenic lines (Figure 3D) when the external K^+^ content was up to 100 μM. The rescued phenotype confirmed the function of HbHAK1 as a high-affinity K^+^ transporter mediating K^+^ uptake under low-K^+^ environment.

**Figure 4:**
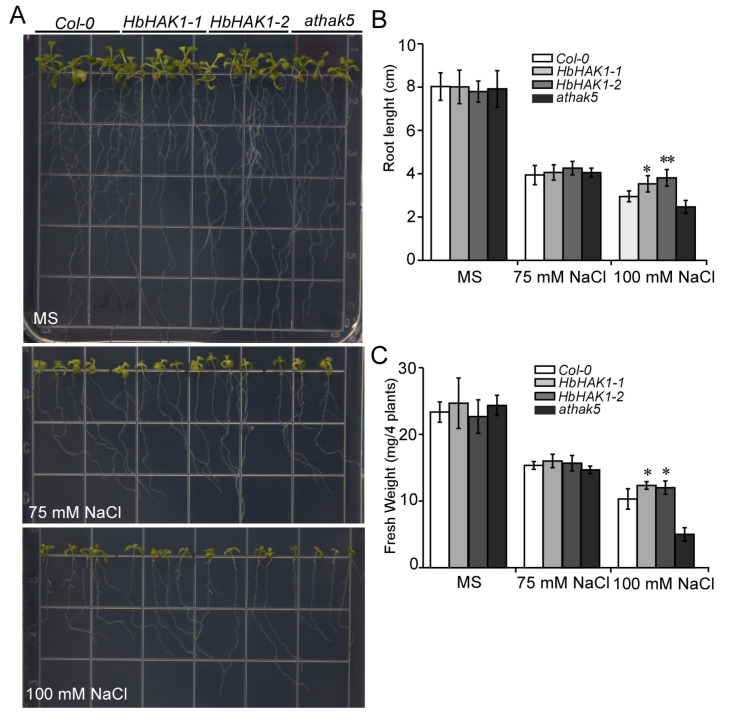
Expression of *HbHAK1* improved the salt tolerance of plants. (**A**) Wild type, two *HbHAK1*-*GFP* transgenic lines in *athak5* (*HbHAK1*-*1* and *HbHAK1*-*2*) and *athak5* grown for 7 days on MS medium and then transferred MS with high NaCl concentration for another 7 days growth; (**B**,**C**) root length (**B**) and fresh weight (**C**) of plants in (**A**). Each bar represents the mean root length of 30 seedlings from three independent experiments in (**B**), and the mean fresh weight (*n* = 3) of 4 seedlings in (**C**). Data represent mean ± SD; Student’s *t*-test was used to identify significant differences at the *p* < 0.05 (*) and *p* < 0.01 (**) level.

Under salt condition, the accumulation of Na^+^ in root cells can depolarize plasma membrane and reduce the driving force for K^+^ uptake. In order to access whether HbHAK1 contributes to salt tolerance, we transferred 3-day-old seedlings of wild type, two *HbHAK1* transgenic lines in *athak5* and *athak5*-mutant to plates with different NaCl content treatment for another 7 days. The two transgenic lines showed better growth under salt conditions than *athak5* and wild type, especially under 100 mM NaCl (Figure 4A). This was further evaluated by measuring the length of primary roots and the fresh weight of plants. Under high-salt conditions, the primary root lengths of *HbHAK1* transgenic lines were obviously longer than wildtype, indicating HbHAK1 can mediate salt tolerance of plants (Figure 4B). The results suggested the improvement of salt tolerance of *Arabidopsis* plants via the expression of *HbHAK1*, possibly depended on enhancing K^+^ uptake under high Na^+^ environment that lead to K^+^ deficiency.

**Figure 5:**
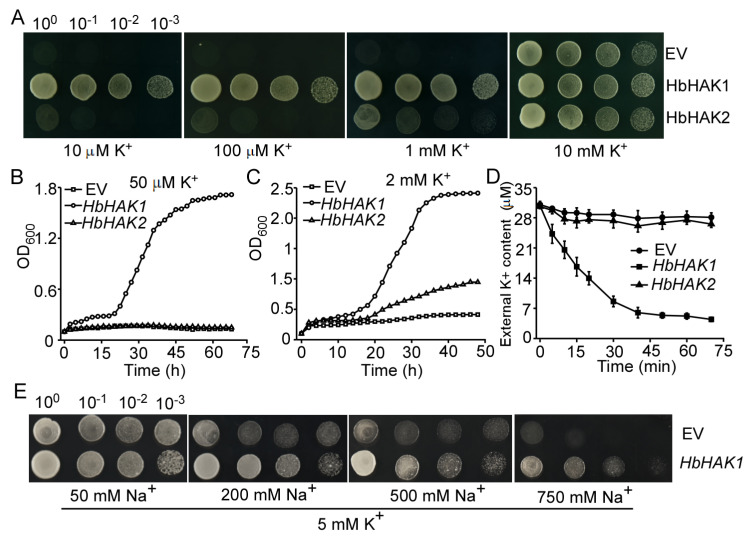
*HbHAK1* and *HbHAK2* complementation assays in yeast Cyl62 stains. (**A**) Growth of the Cy162 strains with p424 (empty vector), p424-*HbHAK1* or p424-*HbHAK2* transformed in solid AP-T medium with various K^+^ concentrations. Drop serial dilutions of each strains were cultured on agar plates; (**B**,**C**) growth curves of the Cy162 strains transformed with p424, p424-*HbHAK1* or p424-*HbHAK2* in liquid AP-T medium with 50 μM K^+^ (**B**) or 1 mM K^+^ (**C**) added; (**D**) K^+^-depletion experiment in the presence of 30-μM K^+^ in liquid AP-T medium. The Cy162 strains transformed with p424, p424-*HbHAK1* or p424-*HbHAK2* were subjected to K^+^ starvation for 4 h prior to the beginning of the experiment. The K^+^ content in the buffer was measured at intervals over a 2 h period. Three independent experiments were carried out and the data represent the mean ± SD; (**E**) growth of Cy162 strains transformed with p424 and p424-*HbHAK1* in solid AP-T medium with various Na^+^ concentrations under 10 mM K^+^.

To identify the hypothesis of K^+^ uptake by HbHAK1, we introduced *HbHAK1* and *HbHAK2* into the yeast Cy162 strains. The Cy162 *trk1*△*trk2*△strains with loss of Trk1 and Trk2 potassium transporters is a perfect system for complementation study [28]. Drop serial dilutions of each strain were cultured on plates in AP-T medium with various K^+^ added and images were captured after 2–3 days growth. When the external K^+^ content was only 10 μM, *HbHAK1* transformed strains could grow well even at 10^−3^ dilution while *HbHAK2* and empty vector transformants were unable to grow. The growths of all the strains were similar at the 5 mM K^+^ content (Figure 5A). Growth curves of the yeast cells in liquid Ap-T medium at different K^+^ concentrations further demonstrated the growth capacity of the *HbHAK1* transformed strains. At 50 μM K^+^ content, the strains expressing *HbHAK1* can grow well and the OD_600_ was up to 1.8 in 60 h, however, the OD_600_ of *HbHAK2* and empty vector transformed strains were still at a low level after 72 h (Figure 5B). When the added K^+^ was reached to 2 mM, the *HbHAK1* transformed strains were strongly growing and the OD_600_ was at 2.0 within 30 h, meanwhile, the OD_600_ of *HbHAK2* transformants was still staying at 1.0 after 48 h (Figure 5C). For the kinetic study of HbHAK1 transporter, we carried out testing medium K^+^ depletion experiment. Under the AP-T medium with 30 μM K^+^ content, Cy162 cells expressing *HbHAK1* showed rapid K^+^ depletion in 20 min, indicating the rapid K absorption by HbHAK1, however, no depletion was observed in Cy162-*HbHAK2* and empty vector transformants (Figure 5D). These results significantly identified the K^+^ uptake function of HbHAK1 under extremely low-K^+^ conditions.

### 2.4. HbHAK1 Strongly Mediated K^+^ Uptake Under Low-K^+^ Conditions or High Na^+^ Condition

To test the Na^+^ influence on HbHAK1, we performed drop serial dilutions experiments in Cy162 system under 5 mM K^+^ conditions with series of gradient Na^+^ added. The results showed that the growth of empty vector transformed strains was obviously inhibited by external Na^+^ added, however, HbHAK1 transformants still had better performance under Na^+^ treatment, even at 750 mM Na^+^ conditions which caused the failed growth of empty vector transformed strains (Figure 5E). To test whether Na^+^ was also the substrate of HbHAK1, we introduced HbHAK1 into B31 *ena1–4△ nha1△* yeast strain in which Na^+^ export pumps are disrupted. AtHAK5 worked as a control (Figure S1). The B31 strain transformed *HbHAK1* showed better growth than *AtHAK5* and empty vector under 5 mM K^+^, however, when 50 mM Na^+^ was added, all the strains exhibited salt sensitivity, suggesting HbHAK1 is not responsible for transporting Na^+^ out of cells. We further tested the effect of Cs^+^ on HbHAK1 and transferred to 20 mM Cs^+^ and 20 mM NH_4_^+^ added plates under 5 mM K^+^ (Figure S2). The result showed the K^+^ transport activity of HbHAK1 was greatly inhibited by Cs^+^ or NH_4_^+^. Therefore, the experiments in Cy162 system indicated the intense K^+^ transport activity of HbHAK1 from extremely low-K^+^ conditions and its strong K^+^ uptake promoted the improvement of salt tolerance; however, it has no effect on reducing Cs^+^ toxicity.

### 2.5. The Amino Acid Residues C170 and R342 of HbHAK1 Determined the Stronger Transport Activity in Low-K^+^ Conditions While A13 Under Salt Stress

**Figure 6:**
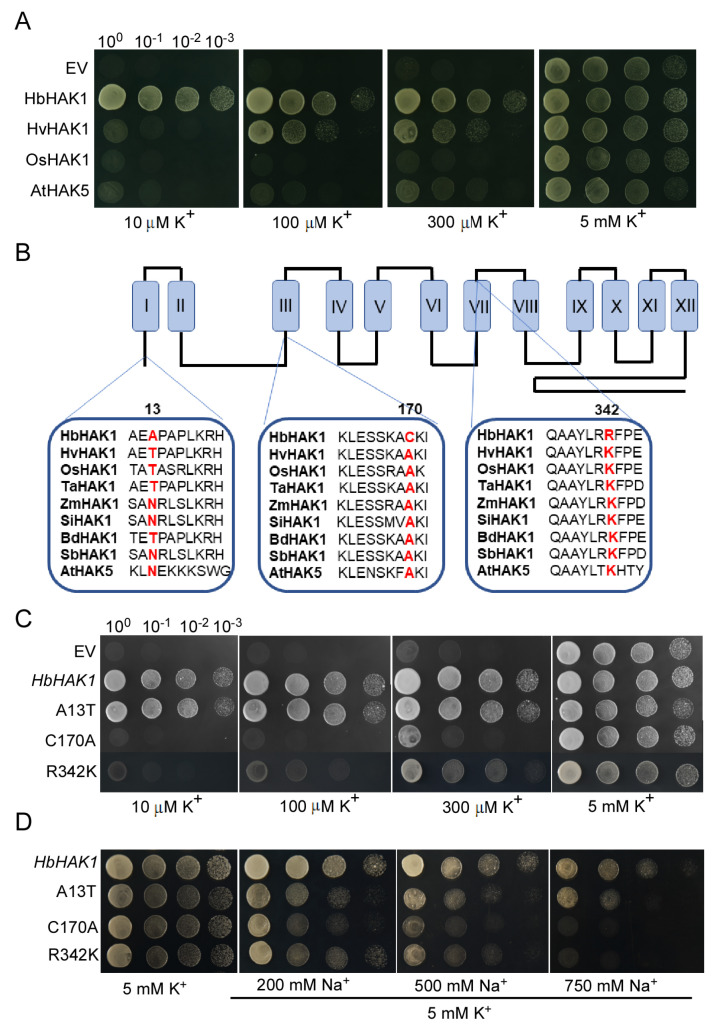
Main amino acids of HbHAK1 affected the transport activity. (**A**) Growth of Cy162 strains transformed with p424, p424-*HbHAK1*, p424-*HvHAK1*, p424-*OsHAK1* and p424-*AtHAK5* in solid AP-T medium with various K^+^ concentrations; (**B**) HbHAK1 topology and the location of three significant amino acid. The proposed model for HbHAK1 protein is shown with 12 transmembrane domains and a long tail in C-terminal. The A13 residue is located in N-terminal. The C170 is located between the second and the third transmembrane domain. The R342 is located in the seventh transmembrane domain; (**C**) growth of Cy162 strains transformed with WT HbHAK1 and the mutants A13T, C170A and R342K in solid AP-T medium with various K^+^ concentrations; (**D**) growth of Cy162 strains transformed with WT HbHAK1 and the mutants A13T, C170A and R342K in solid AP-T medium with various Na+ concentrations under 5 mM KCl.

To compare the transport activity of HbHAK1 with other homologs, we carried out complementation experiment in Cy162 strains transformed with *HbHAK1*, *HvHAK1*, *OsHAK1* and *AtHAK5*. Drop serial dilutions of each strain were cultured on plates in AP-T medium with various K^+^ added and images were captured after 2–3 days growth. The HbHAK1 transformants showed to grow well even at the 10^−3^ dilution under external 10 μM K^+^ concentration, while the HvHAK1, OsHAK1 and AtHAK5 transformed strains were unable to grow. The HvHAK1 transformants grew at 100 μM K^+^ content, and AtHAK5-expressing strains began to grow at 300 μM K^+^ content (Figure 6A). These results confirmed that HbHAK1 had stronger K^+^ transport activity.

Due to the close relationship between *H. vulgare* and *H. brevisubulatum*, we compared the sequences of amino acids between HbHAK1 and HvHAK1 and found that only 15 amino acids were different. To test whether the 15 amino acids affect the transport activity, we carried out site-directed mutation experiment to change each amino acid of HbHAK1 to that of HvHAK1. Colonies transformed with each mutant of *HbHAK1* growing in SD-T selective media were transferred to plates with AP-T medium with different K^+^ added. Drop complementation assays were performed to identify the activity of K^+^ transport. Based on the transporter activity analysis, the mutants of A13T, C170A and R342K were selected. Many HAK1-type proteins in monocotyledonous species with genome published and AtHAK5 in *Arabidopsis* were used to run ClustalW alignment. Notably, the conserved site A170 in cytoplasmic loop in the other species was changed to C170 in HbHAK1, meantime conserved K342 in the seventh transmembrane domain was instead of R342 in HbHAK1 (Figure 6B). Colonies transformed with WT HbHAK1 could grow well under low-K^+^ conditions (10 μM), however, C170A or R342K single mutant transformed strains became unable to grow (Figure 6C). R342K transformants gradually recovered to grow until the K^+^ content was above 100 μM which showed the similar growth with HvHAK1, while C170A transformants were able to grow until the K^+^ concentration was to 300 μM (Figure 6C). A13 was in N-terminal of HbHAK1 and the A13T mutant transformed strain showed greatly similar performance with WT HbHAK1, however, with external Na^+^ increasing, the A13T transformants exhibited weaker growth compared with WT HbHAK1 transformants (Figure 6C,D). The results revealed the importance of the amino acid residues of C170 and R342 for determining K^+^ selecting and transport activity under extremely low-K^+^ conditions, the residue of A13 for enhancing K^+^ uptake under high-salt conditions to improve salt tolerance. Because HbHAK1 was from the wild species of *H. vulgare*, its critical selected amino acid sites provided evidences that these significant natural variant sites may function as the stronger K^+^ transport activity for HbHAK1, implying the key roles of these residues for the function-structure relationship studies of HAK1-type K^+^ transporters.

## 3. Discussion

K^+^ acquisition in plant roots consists of two uptake systems: a high-affinity transporter system (HATS) via H^+^/K^+^ symports at low (<0.2 mM) external K^+^ content and a low-affinity system (LATS) via ion channels at high (>0.5 mM) external K^+^ content. High-salt soil frequently causes K^+^ deficiency, therefore, the HATS in roots dominates K^+^ uptake from soil under saline conditions to maintain a stable cytosol K^+^ concentration (100–150 mM) [4,29]. Since the identification of HvHAK1 in barley [7] and AtHAK5 in *Arabidopsis* [30], HATS is considered to be operated by HAK/KUP/KT transporters [4]. AtHAK5 plays a pivotal role in maintaining high-affinity K+ uptake under saline conditions [31], while different Cluster I members of HAK/KUP/KT family like OsHAK1, OsHAK5 and OsHAK21 coordinately contribute to high-affinity K^+^ transport in K^+^/Na^+^ homeostasis under salt [4,8,9,19]. To survive under saline conditions for plants, maintaining continuous K^+^ acquisition at high external Na^+^ content is crucial for K^+^/Na^+^ homeostasis. The halophytic *H. brevisubulatum* could grow well under heavy saline-alkali land and be used as a saline-tolerant grass for soil improvement in North China [26,32], implying that the *H. brevisubulatum* plant may evolve some distinctive strategies for K^+^ acquisition improvement under saline conditions. The hypothesis was supported by the salt-treated root transcriptome data which showed abundant genes related to potassium ion transport were upregulated [26]. Among these genes, HAK-type transporters were obviously selected, in accordance with the root HATS operating K^+^ uptake under saline conditions.

Through comparing the expression patterns of HAK/KUP/KT proteins between *H. brevisubulatum* and *H. vulgare*, the expression of *HbHAK1* was induced by salt while transcripts of *HvHAK1* was greatly downregulated (Figure 2). We further identified the function of HbHAK1 as a high-affinity K^+^ transporter via rescuing the phenotype of *athak5* in plant growth and salt tolerance (Figure 3 and Figure 4). HAK/KUP/KT transporters could not generate K^+^ currents in cells, thus, their function studies mainly carried out in yeast or bacteria mutants defective for K^+^ uptake like yeast Cy162 strains. Interestingly, the HbHAK1 transformants can grow in the presence of extremely low external K^+^ (10 μM), as the same experiments confirm HvHAK1, OsHAK5, CcHAK1 and CaHAK1 transformants recover the growth at 0.1-mM K^+^ added [12,33,34] and OsHAK1 or AtHAK5 expressing strains can grow at 0.3-mM external K^+^ content [8,10]. The best performance of HbHAK1 in complementation of Cy162 strains indicated its strong K^+^ transport activity and extended the minimum threshold of K^+^ uptake operated by HAK-type transporters.

In fact, because of closed relationship between *H. brevisubulatum* and barley, HbHAK1 and HvHAK1 have only 15 different amino acid residues. We used site-directed mutations in cDNA of HbHAK1 and identified the transport activity of mutants HbHAK1 in Cy162 system. C170A and R342K mutant transformants critically reduced the capacity of strains growth under 10 μM K^+^ conditions which showed even weaker growth compared with HvHAK1 at 0.1 mM K^+^ and 0.3^+^ mM K^+^ added, while A13T mutants dramatically decrease Na^+^ tolerance (Figure 6). C170 and R342 were located at the strictly conserved region of Cluster I proteins in crop species and *Arabidopsis*, which indicated C170 and R342 were located in the core region for K^+^ selectivity (Figure 6). The other identified K^+^ transporter families, such as Shaker channels, KOC channels and HKT transporters, are in charge of putative pore forming region from an ancestor submit of K^+^ channel based on the research about K^+^ channel in animals, which is present in all K^+^ channels among all the kingdom [35,36,37,38]. This region favored the structure–function relationship studies of these K^+^ transporters and deep analysis of their transport activities. However, no such conserved pore forming region is in HAK transporters and HAK-type transporters are absent in animal kingdom, so structure–function relationship studies on HAK family proteins are almost unexplored field and identification of function domain is an interesting work. All previous researches used either random mutagenic PCR or the occurrence of spontaneous mutation of DNA to link the function change. In yeast system, the V336I and R591C mutants of HvHAK1 were revealed to improve K^+^ nutrition and increased Na^+^ tolerance for barley [39]; the L776H and F130S mutants of AtHAK5 improved growth of yeast cells under low-K^+^ and enhanced tolerance to Na^+^ and Cs^+^ in *Arabidopsis* [40,41]; the R443S, L603H, G606E and R443S of PpH AK1 increased Vmax for Rb^+^ absorption in *Physcomitrella patens* [42]. As the *H. brevisubulatum* plants are halophytes and wild species in *Hordeum*, the evolutionarily adaptive sites of C170 and R342 in conserved domain of HbHAK1 were from natural selection which favored improving K acquisition to cope with high-salt stresses. Moreover, A13 site was failed in conserved region, but this site was either T13 or N13 in other plants and the changed A13 in HbHAK1was unique. As the A13 site was in N-terminal of HbHAK1, it particularly led to the changes of Na^+^ sensitivity not Cs^+^ or NH_4_^+^ which suggested A13 site may work as external salt reception site. In conclusion, although many key sites of HAK-type proteins were explored, our knowledge of core region of K^+^ selectivity, external ion sensing and activity regulation is still fragment. The finding of key naturally selective amino acid sites for HAK1-type protein in *H. brevisubulatum* provided significant evidence for exploring the structure function studies of HAK-type proteins and indicated the potential value for crop to promote K^+^ uptake under saline conditions.

## 4. Materials and Methods

### 4.1. Plant Materials and Growth Condition

Seeds of *H. brevisubulatum* in this study used were collected from saline grasslands in Inner Mongolia, China. The methods of the hydroponic culture of the seedlings were described previously [26].

Seeds of *Arabidopsis athak5* stored in lab [27] and Columbia (*Col*-*0*) were plated on MP (Medium lacking Potassium using for plant culture) medium with 0.5% sucrose and 0.8% agar for a week at 21–22 °C with a 16 h/8 h (day/night) photoperiod and 60–70% relative humidity.

### 4.2. Isolation of HAK/KUP/KT Family Genes from H. brevisubulatum Transcriptomic Database

Total RNA was isolated from the root of *H. brevisubulatum* and *H. vulgare* via an RNAprep Pure Plant Kit (Tiangen Biotech Co., Ltd., Beijing China). Deep sequencing was performed by Beijing Ori-Gene Science and Technology Corp., Ltd. (Beijing, PR China). The transcriptome library construction and sequencing data analysis was described in previous research [26]. The raw RNA-seq read data was deposited in http://www.ncbi.nlm.nih.gov/sra/; the accession number is SRP161471.

Protein sequences of HAK/KUP/KT family genes in *Oryza sativa* were obtained from the NCBI website. These sequences were used as a BLAST query in the transcriptomic data to identify putative homologs. 13 HAK/KUP/KT proteins with full length of CDS were obtained and their expression data were also extracted. A phylogenetic tree was constructed in MEGA5.0 software (http://www.megasoftware.net/) using protein sequences of these 13 genes and 27 protein sequences of HAK/KUP/KT family in *Oryza sativa*. The protein sequences of 13 HAK/KUP/KT transporters were list in Figure S3.

### 4.3. HbHAK1 Expression Transgenic Lines in Arabidopsis athak5

*HbHAK1* was constructed into the vector pBASTA, with green fluorescent protein (GFP) expression driven by the CaMV 35S promoter present in the parent plasmid pBI121 [43] using gateway technology, and then transformed into *Arabidopsis athak5* plants using the floral dip method. Transgenic T2 seeds were used for the phenotype analysis. Seeds from the wild type, *athak5* and two different transgenic lines overexpressing *HbHAK1* on an *athak5* background (*HbHAK1*-*1* and *HbHAK1*-*2*) were planted in a seed germination pouch with liquid MP medium containing different concentrations of potassium chloride (KCl) [44]. The Seed germination pouches were placed vertically and photographed after 6 days.

### 4.4. Subcellular Localization of HbHAK1

The cDNA sequences of *HbHAK1* overexpression transgenic T1 generation seeds of *athak5* were germinated and grown for 4 days at 21–22 °C under dark condition. The leaf epidermal cells of the etiolated seedlings were used for microscopic observation. FM4–64 was used to stain the cell plasma membrane. These 4-day seedlings were immersed in 20 μg/mL FM4–64 buffer for 1 min and then washed with water twice. Confocal images were captured using confocal macroscope equipment (Zeiss, Jena, Germany). The fluorescence signals were excited at 488 nm for GFP and 561 for the FM4–64 dye.

### 4.5. Complementation of HbHAK1 in Cy162 Yeast Strains

*HbHAK1* and *HbHAK2* were amplified with special primers and digested with the SpeI/XhoI or EcorⅠ/XhoI restriction enzymes, respectively, and ligated into the yeast expression vector p424 [45]. P424, p424-*HbHAK1*, p424-*HbHAK2*, p424-*HvHAK1*, p424-*AtHAK5*, and p424-*OsHAK1* were transformed into yeast (*Saccharomyces cerevisiae*) strain CY162 *trk1△trk2△* [28] and B31 *ena1*–*4△ nha1△* [46]. The primers used was listed in Supplemental Table S1.

Yeast complementation assays at low K^+^ were performed in solid AP-T (Arginine Phosphate medium lacking Trp) medium, as described previously [39] and supplemented with concentrations of K^+^ ranging from 0.001 to 10 mM, and in the absence or presence of various concentrations of NaCl (50 to 750 mM). For the growth curve, the yeast strains (transformed with p424, p424-*HbHAK1* and p424-*HbHAK2*) were grown in liquid SD-T (minimal Synthetic Defined base with -Trp dropout supplement) medium at 30 ℃ overnight and then transferred to liquid AP-T medium supplemented with different concentration of K^+^ (0.05 or 2 mM) with the same initial OD_600_ of about 0.1. The shaker was adjusted to 200 rpm, and the OD_600_ of the strains was measured every 3 h for three consecutive days of growth. This experiment was repeated in triplicate.

### 4.6. K^+^ Depletion and Measurement of K^+^ Contents in Cy162 Yeast Strains

For K^+^ depletion experiments, yeast cells were grown overnight at 30 °C in liquid SD-T medium and then then transferred to liquid AP-T medium for about 4 h for K starvation. The cells were then suspended in 10 mM 2-(N-morpholino) ethane sulfonic acid (MES) supplemented with 2% glucose and adjusted to pH 6 with Ca(OH)_2_. At time zero, 0.03 mM KCl were added to the medium and the samples were collected at intervals over a 2-h period. K^+^ contents were identified and quantified by atomic emission spectrophotometry using a PerkinElmer Model 2380 spectrophotometer (Waltham Mass, US) [47].

### 4.7. Site-Directed Mutagenesis of HbHAK1

For the changes, the different amino acids of HbHAK1 with HvHAK1, we mutated 15 amino acid residues of HbHAK1 according to the right sequence of HvHAK1 by using the Quick change method with site-directed mutagenesis kit (TransGen Biotech, Beijing, China). The protocol was followed as recommended. The primers used was listed in Supplemental Table S1.

### 4.8. Accession Numbers

The selected 13 genes for the HAK/KUP/KT in *H. brevisubulatum* were exhibited in Figure S3. The gene IDs of coordinate genes in *H. vulgare* can be found in the http://plants.ensembl.org/Hordeum_vulgare/Info/Index, as *HvHAK1* (HORVU2Hr1G071570), *HvHAK2* (HORVU2Hr1G020220), HvHAK7 (HORVU3Hr1G098670), HvHAK9(HORVU2Hr1G018190), HvHAK11 (HORVU2Hr1G098940), HvHAK15 (HORVU2Hr1G099810), HvHAK18 (HORVU5Hr1G090010), HvHAK23 (HORVU5Hr1G059200) and HvHAK25(HORVU6Hr1G073030).

## Figures and Tables

**Table 1:** The list of 13 identified HAK/KUP/KT proteins in *H. brevisubulatum*.

Gene	Protein Length	TMS ^a^	PL ^b^
*HbHAK1*	776	12	plasma membrane
*HbHAK2*	777	13	plasma membrane
*HbHAK5*	708	11	plasma membrane
*HbHAK7*	784	13	plasma membrane
*HbHAK8*	789	12	plasma membrane
*HbHAK9*	781	11	plasma membrane
*HbHAK11*	792	14	plasma membrane
*HbHAK12*	763	14	plasma membrane
*HbHAK16*	799	11	plasma membrane
*HbHAK18*	785	14	plasma membrane
*HbHAK23*	945	12	plasma membrane
*HbHAK25*	769	13	plasma membrane
*HbHAK27*	874	12	plasma membrane

^a^ TMS: number of transmembrane segments posses; ^b^ Localization of HbHAKs protein supported by TargetP.

## Supplementary Materials

Supplementary materials can be found at https://www.mdpi.com/1422-0067/21/15/5292/s1. Figure S1 The growth of B31 strains transformed with the empty vector p424 (EV), p424-HbHAK1 (HbHAK1) or p424-AtHAK5 (AtHAK5) in solid AP-T medium without or with 50 mM Na^+^ under 10 mM K^+^ condition. Figure S2 The growth of Cy162 strains transformed with WT HbHAK1 and the mutants A13T, C170A and R342K in solid AP-T medium under 5 mM K^+^ concentration with 20 mM Na^+^, 20 mM Cs+ or 20 mM NH_4_^+^ added. Figure S3 Multiple alignment of 13 obtained HAK/KUP/KT proteins. Table S1 The primers used in this paper.
