# Supplementary material for: Halophytic Hordeum brevisubulatum HbHAK1 Facilitates Potassium Retention and Contributes to Salt Tolerance
Source: Int J Mol Sci. 2020 Jul 25;21(15):5292. doi: 10.3390/ijms21155292 (PMC7432250; doi:10.3390/ijms21155292)
Supplement: Supplementary file 1 [file ijms-21-05292-s001.zip › Table S1.docx]

**Table S1** Primers used in this study.

| **Primer** | **Restriction sites** | **Primer sequence (5′-3′)** |
| --- | --- | --- |
| *GW-HbHAK1-GFP-F* | *attB1* | GGGGACAAGTTTGTACAAAAAAGCAGGCTTCATGTCGCTGCAAGTCGAGGA |
| *GW-HbHAK1-GFP-R* | *attB2* | GGGGACCACTTTGTACAAGAAAGCTGGGTCTATATCTCGTATGTGATCC |
| *AtActin2-F* |  | GGTGATGGTGTGTCT |
| *AtActin2-R* |  | ACTGAGCACAATGTTAC |
| *RT-HbHAK1-F* |  | AATGGTGATAGGCGATGGAAC |
| *RT-HbHAK1-R* |  | AACCGCTGGACCGAGAAGAG |
| *P424-HbHAK1-F* | SpeI | GACTAGTATGTCGCTGCAAGTCGAGGAC |
| *P424-HbHAK1-R* | XhoI | CCCTCGAGCTATATCTCGTATGTGATCCCGAC |
| *P424-HbHAK2-F* | EcoRI | GGAATTCATGTCTGGACTGTACAATTAC |
| *P424-HbHAK2-R* | XhoI | CCCTCGAGCTAGACGTAGTAGATCATGC |
| *HbHAK1-A13T-F* |  | GACCCGCGGAGCGCGGAGACGCCCGCGCCG |
| *HbHAK1-A13T-R* |  | TCTCCGCGCTCCGCGGGTCCTCGACTTGCA |
| *HbHAK1-C170A-F* |  | TTGAGTCCAGCAAGGCCGCCAAGATTGTGC |
| *HbHAK1-C170A-R* |  | GCGGCCTTGCTGGACTCAAGCTTCTGCTTC |
| *HbHAK1-R342K-F* |  | GGCGGCCTACCTGAGGAAATTCCCAGAGA |
| *HbHAK1-R342K-R* |  | TTCCTCAGGTAGGCCGCCTGCCCGATATA |
| *HbHAK1-E345D-F* |  | CTGAGGAGATTCCCAGACAACGTTGCAAA |
| *HbHAK1-E345D-R* |  | GTCTGGGAATCTCCTCAGGTAGGCCGCCT |
| *HbHAK1-I469M-F* |  | TGGAAGAAGCACGTCATGTTCATCATGCT |
| *HbHAK1-I469M-R* |  | CATGACGTGCTTCTTCCATATGAGCAGCA |
| *HbHAK1-K520R-F* |  | GCACTACGTCCAGGTGAGGAGGTACTGGT |
| *HbHAK1-K520R-R* |  | CTCACCTGGACGTAGTGCCAAGCCGCCAT |
| *HbHAK1-V532I-F* |  | TCGACCACATCGTGCCCATCAGCGAGATG |
| *HbHAK1-V532I-R* |  | TGGGCACGATGTGGTCGAGCTCGTACCAG |
| *HbHAK1-M580I-F* |  | GTGCACTCCATCTTCATCTTCATGTCGAT |
| *HbHAK1-M580I-R* |  | GATGAAGATGGAGTGCACGGACGGTATCT |
| *HbHAK1-L593V-F* |  | TGCCCATCTCGCGCGTGGTGCCCACGGAG |
| *HbHAK1-L593V-R* |  | CCACGCGCGAGATGGGCAGGTGCTTGATC |
| *HbHAK1-A650V-F* |  | GAGAGCGCGTTCGCGCTCGTGCAGGACGAA |
| *HbHAK1-A650V-R* |  | ACGAGCGCGAACGCGCTCTCTTCCTGGATG |
| *HbHAK1-E653Q-F* |  | TTCGCGCTCGCGCAGGACCAAGAGGAGAGC |
| *HbHAK1-E653Q-R* |  | GGTCCTGCGCGAGCGCGAACGCGCTCTCTT |
| *HbHAK1-E661D-F* |  | AGCGGCGGCGCCGGTGATGTTTCGGACGC |
| *HbHAK1-E661D-R* |  | ATCACCGGCGCCGCCGCTCTCCTCTTCGT |
| *HbHAK1-A665--F* |  | CCGGTGAGGTTTCGGACGCCCAGGCGAGGC |
| *HbHAK1-A665--R* |  | GCGTCCGAAACCTCACCGGCGCCGCCGCTC |
| *HbHAK1-Q667L-F* |  | AGGTTTCGGACGCGGCCCTGGCGAGGCCGA |
| *HbHAK1-Q667L-R* |  | AGGGCCGCGTCCGAAACCTCACCGGCGCCG |
| *HbHAK1-N701S-F* |  | ATGAGTTTCCACACGAGCCAGGCGGTGG |
| *HbHAK1-N701S-R* |  | CTCGTGTGGAAACTCATCCTCCCTGAGG |
