# Supplementary figures and images for: Halophytic Hordeum brevisubulatum HbHAK1 Facilitates Potassium Retention and Contributes to Salt Tolerance
Source: Int J Mol Sci. 2020 Jul 25;21(15):5292. doi: 10.3390/ijms21155292 (PMC7432250; doi:10.3390/ijms21155292)

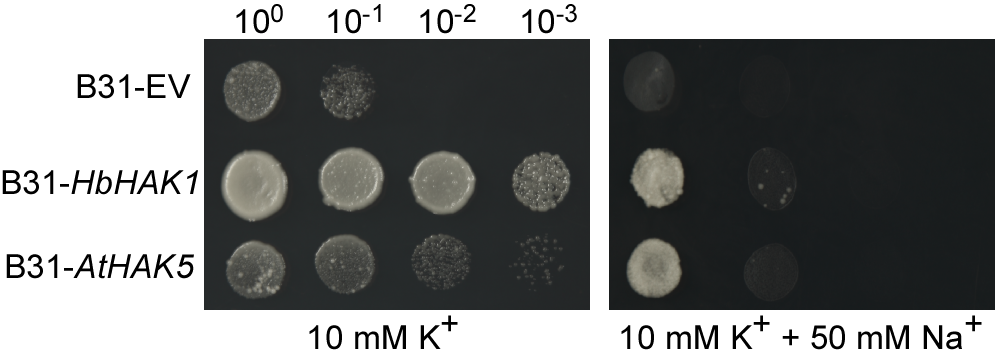

Supplement: Supplementary file 1 [file ijms-21-05292-s001.zip › Figure S1.tif]

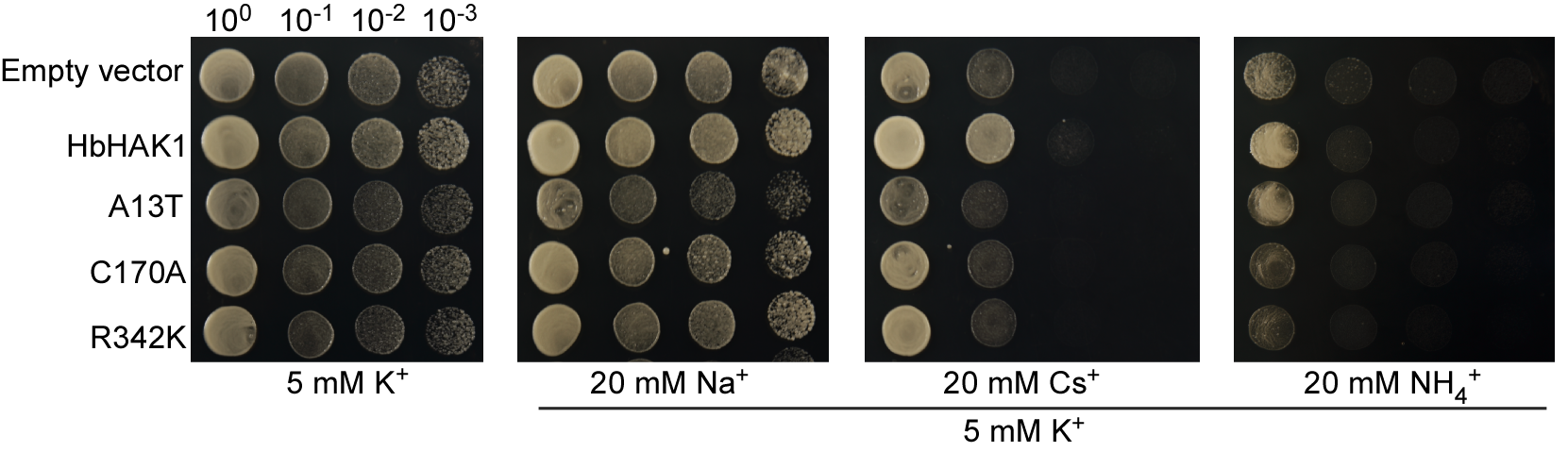

Supplement: Supplementary file 1 [file ijms-21-05292-s001.zip › Figure S2.tif]
